# One-Piece Zirconia Oral Implants for Single Tooth Replacement: Five-Year Results from a Prospective Cohort Study

**DOI:** 10.3390/jfb14020116

**Published:** 2023-02-19

**Authors:** Ralf-Joachim Kohal, Felix Burkhardt, Jerome Chevalier, Sebastian Berthold Maximilian Patzelt, Frank Butz

**Affiliations:** 1 Department of Prosthetic Dentistry, Center for Dental Medicine, Medical Center—University of Freiburg, Faculty of Medicine, University of Freiburg, Hugstetter Str. 55, 79106 Freiburg, Germany; 2 INSA-Lyon, MATEIS Laboratory, University of Lyon, UMR CNRS 5510, 20 Avenue Albert Einstein, CEDEX, 69621 Villeurbanne, France; 3 Private Dental Clinic, Am Dorfplatz 3, 78658 Zimmern ob Rottweil, Germany; 4 Private Dental Clinic, Belchenstrasse 6a, 79189 Bad Krozingen, Germany

**Keywords:** clinical investigation, oral implants, prospective, zirconia

## Abstract

The intention of this 5-year prospective cohort investigation was to clinically and radiographically investigate the outcomes of a one-piece zirconia implant system for single tooth replacement. Sixty-five patients received a total of 66 single-tooth implants. All implants immediately received temporary restorations and were finally restored with all-ceramic crowns. Follow-ups were performed at the prosthetic delivery, after 1, 3, and 5 years. Peri-implant and dental soft-tissue parameters were evaluated and patient-reported outcomes recorded. To monitor peri-implant bone remodelling, standardised radiographs were taken at the implant insertion and at the 1-, 3-, and 5-year follow-ups. In the course of 5 years, 14 implants were lost, resulting in a cumulative implant survival rate of 78.2%. The mean marginal bone loss from the implant insertion to the 5-year follow-up amounted to 1.12 mm. Probing depth, clinical attachment level, bleeding, and plaque index increased over time. In 91.5% of the implants, the papilla index showed levels of 1 or 2, respectively. At the end of the study, the patient satisfaction was higher compared to the pre-treatment measurements. Due to the low survival rate after five years and the noticeably high frequency of advanced bone loss observed in this study, the implant has not met the launch criteria, as it would have not been recommended for routine clinical use.

## 1. Introduction

The clinical application of zirconia oral implants has increased during the past decade [1]. They are regarded as an addendum to titanium implants [2], which still represent the golden standard in oral implantology [3]. The patients’ wish for metal-free restorations, a possible hypersensitivity to titanium, or aspects of aesthetics when titanium might appear inappropriate for certain situations have been stated as reasons for the use of zirconia implants [4]. Zirconia ceramics have a tooth-like colour and exhibit favourable mechanical properties [5]. Its biocompatibility has been proven in a number of animal studies [6,7]. Similar to titanium, zirconia implants, with a micro-rough surface texture, are considered to perform better than implants with a smooth surface [8,9,10]. The capability of zirconia implant systems to withstand masticatory forces in the oral environment was shown in several pre-clinical experiments [11,12,13]. Additionally, several studies showed that zirconia ceramics might be less prone to bacterial adhesion and peri-implant infection than titanium [14,15,16]. Another argument in favour of zirconia implants is the lack of corrosive products (i.e., titanium particles) which may cause potential health hazards [17] or may contribute to the progression of peri-implantitis [18]. However, zirconia has an unfavourable tendency to low thermal degradation [19]. It is not clear yet if this phenomenon has an influence on the long-term success of zirconia as an implant material. Clinical outcomes of zirconia implants are reported to be comparable to titanium implants in short-term and some mid-term studies with observation periods of one and three years [20]. Long-term studies with an observation time of five years or longer are, however, scarce [21]. Therefore, the purpose of this prospective clinical cohort investigation was to evaluate the survival rate and marginal bone remodelling of a one-piece zirconia oral implant when applied for single tooth replacement. The present paper presents the five-year data of the zirconia implant system.

## 2. Materials and Methods

### 2.1. Study Population Clinical Procedure

Patients between 18 and 70 years with no systemic disease requesting the replacement of single missing teeth were acquired for this study. The main inclusion criteria were that the subjects were between 18 and 70 years old, had to be in need of one implant for single-tooth replacement, and were systemically healthy. In addition, sufficient bone volume had to be present in the prospective implant regions. The participants had to have a stable occlusal relationship and no parafunctional habits. The implant sites had to be free of infection and tooth remnants. Main exclusion criteria were alcohol or drug abuse or general health conditions that did not allow a surgical procedure (e.g., bone metabolism disorder). Local contraindications were, for example, tumours and ulcers. Written informed consent was obtained from all subjects. The study protocol was approved by the local ethics committee (investigation number: 337/04; University Clinics Freiburg, Freiburg, Germany). Prior to surgery, prospective implant sites were evaluated with cone beam computed tomography (Newtom 3G; Newtom, Marburg, Germany). Conical, one-piece implants made of yttria-stabilised tetragonal zirconia polycrystal (y-TZP) with a moderately rough surface were used (Nobel Biocare AB, Gothenburg, Sweden). The implant was never commercially released due to failure to meet the launch criteria, as validated by our study. The design of the ceramic implant was similar to the one-piece NobelDirect™ titanium implant (Nobel Biocare). To improve osseointegration, Nobel Biocare introduced a technology leading to a porous surface at the surface of zirconia implants. The porous surface was deposited on already-sintered implants, by coating the endosseous part with a slurry containing zirconia powder and a pore former (patent application SE03022539-2). A second sintering of the implants yielded to the burn-off of the pore former and to a porous surface, with a thickness of 15 µm and a Sa-value of 1.24 µm [22,23]. This rough and micro-porous surface was referred to as “ZiUnite**^®^**”.

From one day before until 3 days after implant placement, patients were provided with Clindamycin 300 mg three times a day. Pain control was administered with Ibuprofen (400 mg). Patients were instructed to take a single dose 1 h prior to surgery and use analgesics postoperatively as necessary. Implants were either placed immediately after tooth extraction or in healed sites. In healed sites, either a flapless procedure with a punch was performed or a full thickness flap was elevated. Subsequently, osteotomies were drilled following the manufacturers protocol and the implants were placed. Finally, implant abutments were slightly prepared for the immediate restoration with relined eggshell temporaries. To avoid excessive forces during the healing period, centric and eccentric contacts were removed from the temporary. Customised intraoral X-ray film holders were used to take standardised radiographs. After the surgical intervention, the patients were instructed to rinse with a 0.2% chlorhexidine solution and not to brush the surgical site for 1 week. After one week, wounds were inspected and sutures were removed. After a healing period of 2 months in the mandibles and 4 months in the maxillae, the implants were definitively restored with all-ceramic single crowns. Conventional impressions were taken, and all-ceramic crowns consisting of a zirconia framework (Procera) and a glass-ceramic veneering (NobelRondo, both Nobel Biocare) were produced and finally cemented with a glass-ionomer cement (Ketac Cem, 3M Espe, Neuss, Germany).

### 2.2. Clinical and Radiographic Assessment

Follow-ups (Appendix A and Appendix B) were performed after 1, 3, and 5 years, including assessment of the papilla index (PI) according to Jemt [24], the probing depth (PD), the clinical attachment loss (CAL), the modified bleeding index (mBI), and the modified plaque index (mPI); the two last indices were according to Mombelli et al. (1987) [25]. The patient reported outcome measurements (PROMs) “function, aesthetics and appearance, sense, speech, and self-esteem” were assessed using a visual analogue scale (VAS). This is a measurement tool for subjective characteristics which cannot be directly measured. The customised film holders were used to take standardised radiographs to monitor bone remodelling over time. For the measurement of bone remodelling over time, the radiographs were calibrated using the known width of the base of the abutment part of the ceramic implants. The lower edge of the implant abutment part was used as the reference point for the measurements (Figure 1a). An independent radiologist at the University of Gothenburg, Sweden, examined all radiographs. Implant success grading as proposed by Östman and co-workers [26] was slightly modified and defined as grade I for implants with no clinical and radiographic signs of pathology showing ≤ 2 mm bone resorption. Success grade II was assigned to implants with no clinical and radiographic signs of pathology and ≤3 mm bone resorption. At the follow-ups, patients were screened for biological complications and other adverse events as well.

### 2.3. Statistical Analysis

For the clinical variables (PD, CAL, mBI, mPI), means and standard deviations were calculated. Subsequently, the values for the implants were compared to the values found for the neighbouring teeth using the Mann–Whitney U-test. The Wilcoxon Signed Ranks test (for PD and CAL) and the Sign test (for mBl and mPl) were applied for the assessment of the changes in the clinical variables over time. Implant cumulative survival rates were calculated using the actuarial life table analysis [27]. Bone remodelling/loss results were presented descriptively using means and standard deviations. The associations of bone level changes as well as cumulative survival rates with different baseline parameters (e.g., jaw type, bone grafting, insertion torque) were evaluated using univariate analyses. For assessing a relationship with ordered categorical and continuous baseline variables, the Spearman correlation coefficient was used. The effects of dichotomous baseline variables on bone remodelling were evaluated using the Mann–Whitney U-test. The significance tests were two-tailed and conducted at a level of statistical significance level of *p* ≤ 0.05. All calculations were performed with a statistical software (SPSS, version 20.0, IBM Corporation; Armonk, NY, USA).

## 3. Results

The majority of the implants was placed in the age group from 31 to 50 years (Table 1). Forty-seven of the 66 implants were placed in posterior mandibular sites (33 in molar areas), 13 in posterior maxillary sites (nine in premolar areas), 5 in anterior maxillary sites, and 1 implant was placed in the anterior mandible (position 33). Forty-five 5.0 mm diameter implants and twenty-one 4.3 mm diameter implants were inserted. The length distribution of the applied implants can be depicted from Table 2. Minor bone grafting procedures were performed in 23 cases. In total, 61 implants were placed in healed sites, whereas only 5 implants were placed in extraction sockets. In a major share of the surgeries, flaps without releasing incision were raised (36) or flapless surgery with a punch (18) was carried out. Mono-cortical anchorage was predominant (57), while bi-cortical anchorage was only achieved in six cases. Insertion torque was less than 35 Ncm in 4 cases, between 35 and 45 Ncm in 38 cases, and more than 45 Ncm in 17 cases.

In total, 62 out of the 65 patients received their permanent all-ceramic crowns. Three implants (upper and lower premolar, upper molar) were lost before the restorative procedures (Table 3). These implants did not osseointegrate and were found mobile at the time of their removal. Of the 62 finally restored patients, 61 could be seen at the 1-year follow-up. Due to business reasons, one patient moved away and could not be contacted anymore. This patient was, therefore, counted as a drop-out. Between the 1-year and 3-year follow-up, another three implants in three patients replacing two mandibular molars and a mandibular premolar were removed because of increased peri-implant bone loss. Up to the 3-year follow-up, two more patients had to be counted as drop-outs: one patient did not attend because of time conflicts and another patient moved without leaving a new address. Therefore, only 56 patients out of the remaining 58 patients could be evaluated at the 3-year follow-up. At the 5-year follow-up, 48 patients could be evaluated. A further patient moved and could not be located anymore. Between the 3-year and 5-year evaluations, eight implants (one lower premolar and seven lower molars) were lost. Seven implants had to be removed because of peri-implant infection and one implant fractured. However, it had also a history of peri-implantitis. Thirteen patients with fourteen implants (one patient received two single implants) were withdrawn because of implant loss during the course of the study, and four patients moved/quit and could not be seen anymore (Table 3). The 5-year cumulative survival rate was calculated to 78.0% for this one-piece zirconia oral implant (Table 4). Univariate as well as multivariate analyses did not show that one single factor or a combination of factors influenced the cumulative survival rate of the investigated implants (all *p*-values > 0.05) (Table 5).

### 3.1. Evaluation of Clinical Parameters (Figure 2)

The probing depth (PD) decreased at implant and tooth sites from prosthesis insertion (PI) until the 1-year follow-up (implants: from 2.75 mm to 2.35 mm; teeth: from 2.07 mm to 1.94 mm). The decrease for both over time as well as the differences between implants and teeth at both time points were statistically significantly different (*p* < 0.05). At implants and at teeth, the PD increased to the 5-year follow-up to 3.84 mm (implants) and 2.61 mm (teeth). Again, the increase for implants and teeth over time as well as the difference between both at all time points was statistically significantly different (*p* < 0.05). The clinical attachment level (CAL) showed a similar trend for the implants. CAL decreased from 2.9 mm to 2.71 from PI to 1 year (*p* = 0.215), whereas it slightly increased for the teeth (from 2.4 mm to 2.48 mm; *p* = 0.448). The CAL for implants and teeth, however, showed a continuous increase until the 5-year follow-up, which was significant (5-year implants: 3.98 mm, *p* = 0.000; 5-year teeth: 3.22 mm, *p* = 0.000). The differences between implants and teeth were statistically significant at all time points. At implants as well as at teeth, the mBI slightly decreased from PI (implants: 0.36; teeth: 0.23) to the 1-year follow-up (implants: 0.23; teeth: 0.19; all comparisons; *p* > 0.05). A significant increase until the 5-year follow-up was observed at implants (0.82, *p* = 0.000) and at teeth (0.48, *p* = 0.002), the difference between implants and teeth being significant (*p* = 0.001). In disparity to PD, CAL, and mBI, the mean values for the mPI, were generally higher for teeth than for implants. Both objects of evaluation showed a decrease of mPI from PI (implants: 0.37, teeth: 0.47) to the 1-year follow-up (implants: 0.10, teeth: 0.28; *p* = 0.000). The decrease was in both groups statistically significantly different (*p* < 0.05). Up to the 5-year follow-up, the mPI increased significantly for the implants (0.65; *p* = 0.021) and teeth (0.81; *p* = 0.000). At five years, the difference between implants and teeth was significant (*p* = 0.008). The average papilla index score increased from PI (1.15) to the 3-year follow-up (2.16) with a decrease to the 5-year follow-up (1.64) (Figure 3).

**Figure 2:**
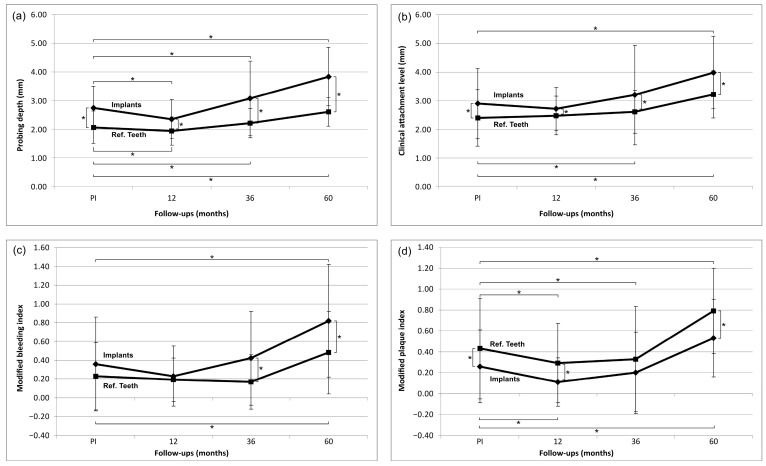
The development of the clinical parameters from prosthesis insertion (PI) to the 5-year follow-up. Data shown as the mean *±* standard deviation (SD). * indicate *p ≤* 0.05. Implants: n_(PI)_ = 63; n_(1-year)_ = 62; n_(3-years)_ = 56; n_(5-years)_ = 48. Reference teeth: n_(PI)_ = 113; n_(1-year)_ = 110; n_(3-years)_ = 103; n_(5-years)_ = 89. (**a**) Probing depth measurement, (**b**) Clinical attachment level measurement, (**c**) Modified bleeding index, (**d**) Modified plaque index.

### 3.2. Biological Complications

Biological complications were seen during the investigation (Table 6). Peri-implant infections were treated following the C.I.S.T. protocol [28].

### 3.3. Marginal Bone Remodelling

**Figure 1:**
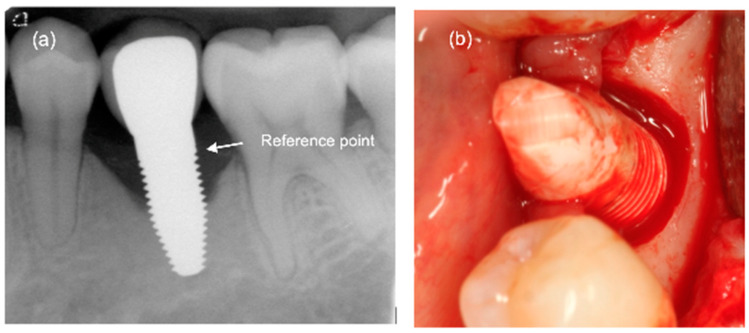
(**a**) Exemplary radiograph at the 5-year follow-up depicting a single-tooth implant with a distinct vertical defect at the mesial and distal aspect of the implant. The lower corner of the straight cylindrical implant part was used as reference point for bone level calculations. (**b**) Clinical situation of the same implant prior to removal.

**Figure 3:**
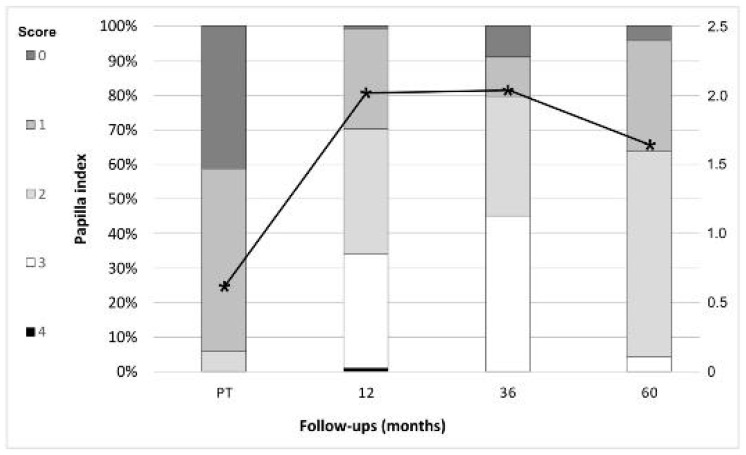
Papilla score distribution (bar graph, left axis) and average score (line graph, right axis) at the different follow-up times. * indicates the average papilla index score.

From implant insertion (II) to PI, the mean marginal bone loss was 1.13 mm and from II to the 1-year follow-up it was 1.31 mm. A bone loss of 1.45 mm between II and the 3-year follow-up and of 1.12 mm between II and the 5-year follow-up was found. The results indicate that there was a slight further increase in bone loss from the 1-year to the 3-year follow-up but a decrease in bone loss from the 3-year to the 5-year follow-up (Table 7). At the 5-year follow-up, at 11 of 41 eligible implants (27%), a marginal bone loss of more than 2 mm was detected. Of these 11 implants, 5 demonstrated more than 3 mm of bone loss. This resulted in a 73% success grade I and to 88% success grade II after 5 years [26]. An exemplary radiograph and clinical picture show the typical bone loss pattern (Figure 1). Regarding any influence or correlation of baseline parameters, the performed univariate analysis did not disclose any effect or correlation of these parameters onto the bone remodelling/loss from implant insertion to the 5-year follow-up (Table 8).

### 3.4. Patient Assessment: Patient-Reported Outcome Measures (PROMs)

**Figure 4:**
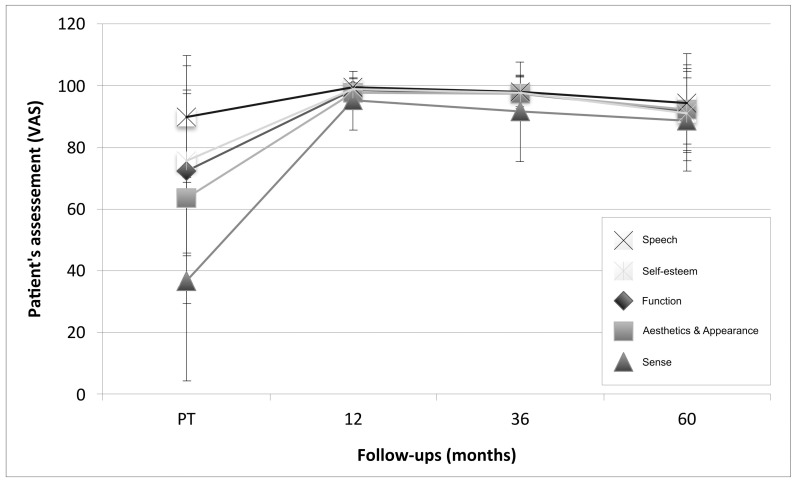
Patient’s assessment (speech, self-esteem, function, aesthetics and appearance, and sense) registered on a VAS scale from 0 to 100%, where 0% indicates a poor condition and 100% an excellent condition. Data shown as the mean *±* standard deviation (SD).

Compared to the pre-treatment situation (36.6%–90.0%), all assessments (function, aesthetics, sense = feels like my own tooth, speech, self-esteem) revealed improvements of the average VAS values at the 5-year follow-up examination (function: from 72.2 to 91.8; aesthetics: from 63.5 to 92.4; sense: from 36.6 to 88.5; speech: from 90.0 to 94.4; self-esteem: from 75.6 to 91.2). The largest change over time was found for the patients’ perception of sense (46.9%) and the lowest for speech (6.5%) (Figure 4).

## 4. Discussion

In the present study, we consecutively report the 5-year results of our preliminary studies [29,30] on moderately rough surface, one-piece, immediately provisionalised, single tooth Y-TZP implants.

The present investigation showed an implant survival rate of 78.2%. In a different study on immediately loaded one-piece single-tooth Y-TZP implants (n = 32), the authors reported on the survival rate after 4.3 to 6 years of 96.8% [31]. In addition, Balmer et al. (2020) evaluated 71 Y-TZP implants (49 single-tooth implants, 22 implants supporting 3-unit fixed dental prostheses; FDPs) with a mean observation time of 5.6 years and a survival rate of 98.4% [32]. Kohal et al. (2020) found a cumulative 5-year survival rate of 94.3% for 53 alumina-toughened zirconia one-piece implants including 27 single-tooth implants and 26 implants supporting 3-unit FDPs [33]. Lorenz et al. (2019) analysed 83 Y-TZP implants supporting either single crowns or FDPs after an observation time of 7.8 years. The survival rate was 100% [34]. Two meta-analyses reported on a 1-year survival rate of ceramic oral implants of 98.3% and 95.6% [35]. The former one calculated the survival rate after two years with 97.2%. Meta-analyses with an observation period of 5 and more years are not available yet. In comparison, the implant survival rate of the present study was about 20% lower.

When focussing on the marginal bone loss (MBL), in the present study, the mean marginal bone loss decreased from the 3-year follow-up (1.45 mm) to the 5-year follow-up (1.12 mm). This obvious gain in bone, however, was due to the removal of implants with high bone loss due to peri-implant infection—if still in situ, they would have increased the MBL calculations significantly. Nevertheless, the 5-year MBL result was in the magnitude of other investigations. Grassi et al. (2015) reported on a mean MBL of 1.23 mm after 4.3 to 6 years [31], and Lorenz et al. (2019) observed a MBL of 1.2 mm after 7.8 years [34]. Lower MBLs were found by Balmer et al. (2020) with 0.7 mm after 5.6 years [32] and Kohal et al. (2020) with 0.81 mm after 5 years [33]. Meta-analyses on marginal bone loss are only available for short term periods of about up to one year and revealed a mean MBL of 0.7 mm [36] and 0.79 mm [35]. However, the high frequency (27%) of implants with bone loss of more than 2 mm is remarkable. In the study by Grassi et al. (2015), only one implant showed a bone loss of more than 2 mm after 4.3 to 6 years [31]. A similar observation was made by Balmer et al. (2020): only one implant (2%) lost more than 2 mm of bone in 5.6 years [32]. A frequency for bone loss of more than 2 mm after 5 years of 8.35% (four implants) was found in the study by Kohal et al. (2020) [33].

Probing depth (PD) and clinical attachment loss (CAL) increased over 5 years with statistical significance for implants and the adjacent teeth. The fact that at the 5-year follow-up, PD and CAL were statistically significantly higher around implants than around the teeth seems to be a common finding [37,38,39]. The increase of PD mean values from prothesis insertion to the 5-year follow-up was noticeable and with 1.09 mm for PD (2.75 ± 0.75 mm to 3.84 ± 1.02 mm), twice as high around implants than around teeth (from 2.07 ± 0.57 mm to 2.61 ± 0.5 mm). Similar results were seen for the CAL. This increase can be explained with marginal bone loss occurring physiologically, but also due to the inflammatory processes. In the study by Balmer et al. (2020), the PD around implants also increased over time from 2.7 mm at 0 months to 3.3 mm at 5 years [32]. Similar findings were reported by Kohal et al. (2020) with an increase of PD from prosthesis insertion (2.67 mm) to the 5-year follow-up (3.27 mm) [33]. The latter two studies did not report on peri-implant inflammatory processes, leading to excessive MBL.

Although more plaque was found around teeth than around implants, the mBI was higher for implants than for teeth. This apparent contradiction could be explained by the fact that the periodontal probe penetrates more easily into the connective tissue at implant sites than at tooth sites. The structural differences in the supracrestal region of teeth with those of peri-implant mucosa—a denser mucosal seal of the gingival vs. the peri-implant tissues—could explain the difference [40].

Patient-reported outcome measures (PROMs) are seldomly reported in clinical implant research. We found that from pre-treatment to the 1-year follow-up, all PROMs increased to levels of more than 95%. The mean increase ranged from 10.1% for speech to 58.6% for sense. Thus, a very positive effect of the implant treatment was apparent. Subsequently, from the 1-year follow-up to the 5-year follow-up, a slight decrease was observed within a range of 5.1% for speech and 7.7% for self-esteem. This decrease has to be attributed to the increasing number of clinically compromised implants which have led the participants to award lower scores. Additionally, the 13 patients who lost their implants during the study were not seen at the follow-ups after their implant losses and could, therefore, not be questioned for their satisfaction. It is possible that the decrease of the scores would have been more pronounced if these patients had also been included.

As in our previous reports, a relationship between bone loss and the evaluated baseline parameters (e.g., jaw type, implant position, bone quality and quantity, implant diameter and length, etc.) could not be detected [29,30]. Therefore, we can only speculate upon the causes for the considerably high amount of bone loss and peri-implant infection around this one-piece zirconia implant. Failed implants from this study were removed with a trephine burr and histologically analysed [41]. The osseointegration patterns were not found to be different from those around titanium implants. Combining these clinical histological results with the results from animal studies investigating similar implant materials and surfaces [10,22] leads to the conclusion that there is no evidence for a lack of osteoconductive potential that might explain the increased bone loss. The design of the zirconia implant in the present investigation was similar to the design of the NobelDirect implant. There were different clinical and radiographic outcomes of that implant system. Some authors reported positive results regarding bone loss and implant survival [42,43]. However, in other investigations, an extensive bone loss—especially in combination with immediate loading—was observed [26,44,45,46,47,48]. The macrogeometry and threads design might have also been responsible for performing a high pressure onto the crestal part of the bone during implant placement in this study. In addition, a rough surface is advantageous for a good bone-implant integration but is also prone to accelerated bacterial colonisation if exposed to the oral environment. In an in vitro study, an increased biofilm formation on the ZiUnite**^®^** surface was observed [49]. Thus, the combination of both crestal bone loss as a consequence of high pressure during implant insertion and subsequently good conditions for biofilm establishment could have favoured the development of bone loss/peri-implantitis in some or all of the 14 cases listed [50].

**Figure 5:**
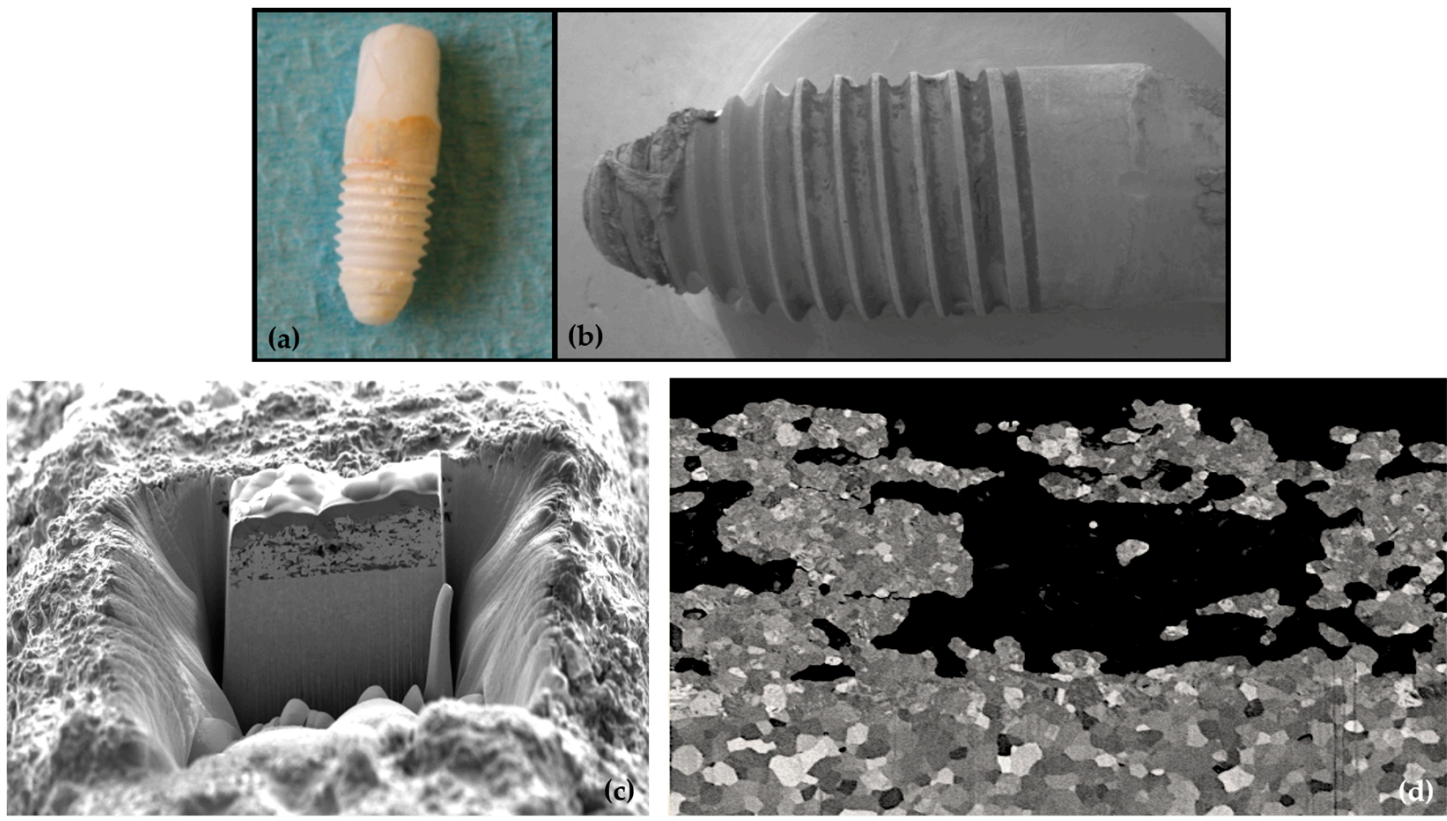
Top: Optical (**a**) and Scanning Electron Image (**b**) of an implant removed after 37 months. Bottom: Focused Ion Beam trench performed at the surface to investigate potential tetragonal to monoclinic transformation of the coating (**c**) and Scanning Electron Microscopy image of the trench (**d**) showing a typical contrast due to the t-m transformation and few microcracks (highlighted with the white arrows). Adapted from C. Sanon’s PhD thesis [51].

Moreover, the surface integrity of the investigated implant was found to be compromised. Cristallographic tetragonal-to-monoclinic (t-m) transformation of the porous layer was observed at an accelerated rate compared to conventional bulk zirconia. This was confirmed by a deep microstructural evaluation of explants of the present study, which exhibited an important transformation rate, after only few months in vivo, associated with micro-cracking [51]. It may be assumed that the micro-cracks could grow during mastication, potentially leading to a partial delamination and loss of the coating. Such effects might have also occurred in our study, with negative consequences for the clinical outcome. Figure 5 shows a picture of an implant removed after 37 months and a Focused Ion Beam trench was made at the surface to investigate the transformation/degradation of the ZiUnite**^®^** surface. The Scanning Electron image shows an extensive transformation of the coating, revealed by a peculiar contrast of the grains in comparison with the bulk underneath. Micro-cracks in the coating were observed on the different cross sections made on several explants. The transformation was confirmed by X-ray diffraction conducted on the endosseous parts of the explant that revealed a monoclinic content of 50% for the implant shown in Figure 5.

Such an extended (and abnormal) t-m transformation after a short duration is clearly a matter of concern and may explain in part the loss of osseointegration of these implants after few years.

Heat development during implant bed preparation, excessive forces during the healing phase, and entrapment of cement are further potential reasons for bone loss around immediately provisionalised, one-piece zirconia implants. These issues also apply to other ceramic implants placed and restored following comparable protocols [31,32,33]. These investigations, however, reported higher survival rates and a lower number of implants with progressive bone loss. Therefore, it may be considered as questionable whether the mentioned aspects have played a decisive role in the present investigation.

Possible limitations of the present investigation were the non-standardised surgical techniques. Both flaps without releasing incision and flapless surgery with a punch were applied. In addition, implants were placed directly after extraction and minor bone grafting was performed in some cases. However, the different analyses (univariate, multivariate) did not show that the different surgical techniques (and other baseline factors) influenced marginal bone loss or the cumulative survival rates after 5 years.

Based on the findings of the present study, the investigated implant has never been released and made commercially available. Nevertheless, the obtained data are essential to fight the tendency to withhold negative results and to reduce the bias that might be inserted into the research dissemination [52].

## 5. Conclusions

The survival rate of the one-piece zirconia implant from the present investigation is inferior to the rates reported of other one-piece zirconia or two-piece titanium implants. Bone remodelling analysis revealed high frequencies of bone loss > 2 mm. A direct relationship between possible confounding factors and the comparably low outcome of this ceramic implant system could not be detected. However, since the specific tapered implant design and the applied surface roughening method (ZiUnite**^®^**) are unique to this implant as compared to other systems, the reason for the poor performance is likely to be associated to these two factors. In particular, the rapid degradation of the surface by ageing might be of concern, since it may have accelerated the loss of bone integration. Consequently, the ZiUnite**^®^** implant was not made commercially available; nevertheless, the data of this ongoing study—even if negative—might be considered to enhance the knowledge of clinical long-term zirconia implant behaviour.

## Figures and Tables

**Table 1:** Patients’ age distribution at implant surgery.

	Number of Patients	%
18–30 years	16	24
31–40 years	21	32
41–50 years	17	26
51–70 years	11	17
Total	65	100

**Table 2:** Implant lengths and diameters.

		Upper Jaw		Lower Jaw	
Diameter	Length	Placed	Failed	Placed	Failed
Regular platform, ∅4.3 mm	10 mm	1	0	10	2
	13 mm	1	0	6	2
	16 mm	3	0	0	0
	Total	5	0	16	4
Wide platform, ∅5.0 mm	10 mm	1	0	9	3
	13 mm	9	2	18	5
	16 mm	3	0	5	0
	Total	13	2	32	12

**Table 3:** Status of patients’ follow-up from implant insertion to the 5-year follow-up.

	Implant Insertion	1 Year	3 Years	5 Years
Followed patients	65	61	56	48
Patients with failed Implants	0	3	6	13
Missing forms	0	1	3	4
Total	65	65	65	65

**Table 4:** Life table analysis.

Time Period	Total Implants	Failed Implants	Missing Forms	Cumulative Survival Rate (%)
Insertion to 1 year	66	3	1	95.5
1 year to 3 years	62	3	3	90.8
3 years to 5 years	57	8	4	78.2
5 years	48			

**Table 5:** Cumulative survival rates and 95% confidence intervals for subgroups at the 5-year follow-up.

Cumulative Survival Rate		
	%	95% CI
**Jaw type**		
Maxilla	88.89	62.42–97.10
Mandible	74.23	59.08–84.47
**Ant-Post**		
Anterior	100.00	-
Posterior	75.99	62.82–85.03
**Position**		
Posterior Mandible	73.66	58.28–84.11
Other positions	89.47	64.08–97.26
**Smoking**		
No	78.22	65.43–86.74
Yes	75.00	12.79–96.05
**Bruxism before treatment**		
No	76.79	63.95–88.55
Yes	100.00	
**Bone quality**		
1	100.00	-
2–3	77.61	65.12–86.10
**Bone quantity**		
A	81.57	65.13–90.77
B	71.58	49.41–85.33
C	100.00	-
D	-	-
**Platform**		
RP	80.67	56.31–92.28
WP	76.65	60.91–86.71
**Implant length**		
10 mm	75.63	50.95–89.08
13 mm	73.20	54.79–85.07
16 mm	100.00	-
**Flap design**		
No flap	100.00	-
Punch	82.96	55.92–94.18
Flap	72.92	56.43–84.00
**Site**		
Immediate	100.00	-
Healed	76.39	63.39–85.29
**Bone grafting**		
No	75.92	59.85–86.26
Yes	82.13	59.03–92.91
**Insertion torque**		
≤45	79.93	63.81–89.43
>45	75.89	51.39–89.20

**Table 6:** Biological complications/Adverse events.

	Insertion to 1 Year	1 Year to 3 Years	3 Years to 5 Years	Total
Pus	-	12	23 (9)	35 (9)
Plaque	-	-	1	1 (0)
Peri-implantitis	-	3	11 (3)	14 (3)

Number within parenthesis ( ) represents recurring adverse event.

**Table 7:** Bone remodelling data from all available radiographs: negative numbers indicate bone loss.

	Implant Insertion to Prosthesis Insertion		Implant Insertion to 1 Year Follow-Up		Implant Insertion to 3 Year Follow-Up		Implant Insertion to 5 Year Follow-Up	
Number	59		56		55		41	
Mean Value	−1.13 mm		−1.31 mm		−1.45 mm		−1.12 mm	
SD	1.47 mm		1.49 mm		1.96 mm		1.83 mm	
	n	%	n	%	n	%	n	%
>0 mm	8	14	7	13	12	22	8	20
0 mm	1	2	0	0	1	2	1	2
−0.1–−1.0 mm	23	39	20	36	13	24	12	29
−1.1–−2.0 mm	11	19	10	18	10	18	9	22
−2.1–−3.0 mm	9	15	11	20	7	13	6	15
−3.1–−4.0 mm	3	5	5	9	7	13	2	5
< −4.0 mm	4	7	3	5	5	9	3	7

**Table 8:** Univariate analysis of marginal bone loss from implant insertion to the 5-year follow-up.

			Difference		Correlation	
	Implants ^a^	Mean (SD)	95% CI	*p* Value	r	*p* Value
**Jaw type**						
Maxilla	9	−0.63 (2.2)	−1.1 to 2.4	0.40		
Mandible	32	−1.26 (1.7)				
**Ant-Post**						
Anterior	3	−1.45 (3.9)	−9.8 to 9.1	0.58		
Posterior	38	−1.10 (1.7)				
**Position**						
Posterior Mandible	31	−1.21 (1.7)	−2.0 to 1.3	0.65		
Other positions	10	−0.84 (2.2)				
**Smoking**						
No	40	−1.02 (1.7)	0.59	7.67		
Yes	1	−5.15 (-)				
**Bruxism before treatment**						
No	38	−1.24 (1.9)	−3.6 to 0.3	0.10		
Yes	3	−0.42 (1.0)				
**Bone quality**						
1	1	−1.85 (-)	−4.5 to 3.0	0.53		
2–3	40	−1.10 (1.8)				
**Bone quantity**						
A	25	−1.20			0.15	0.61
B	15	−1.27				
C	1	2.90				
D	0	-				
**Bone level at placement**	41				−0.36	0.36
**Platform**						
RP	13	−0.60 (1.7)	−0.5 to 2.0	0.09		
WP	28	−1.36 (1.9)				
**Implant length**						
10 mm	13	−1.03 (1.5)			−0.15	0.28
13 mm	20	−0.86 (1.6)				
16 mm	8	−1.93 (2.7)				
**Flap design**						
No flap	3	−3.55 (2.2)			0.29	0.16
Punch	10	−0.97 (2.0)				
Flap	28	−0.92 (1.6)				
**Site**						
Immediate	2	−0.80 (5.2)	−45.7 to 46.3	0.95		
Healed	39	−1.14 (1.7)				
**Bone grafting**						
No	26	−1.10 (1.7)	−1.2 to 1.4	0.88		
Yes	15	−1.16 (2.1)				
**Insertion torque**						
≤45	27 ^b^	−1.11 (1.7)	−0.9 to 1.6	0.60		
>45	13 ^b^	−1.46 (1.8)				

SD, standard deviation; CI, confidence interval. ^a^ The sum of implants is 41, for which matching radiographs were available at baseline and the 5-year follow-up. ^b^ The sum of implants regarding insertion torque is 40 because for one implant, there was no reading available.

**Figure A1:**
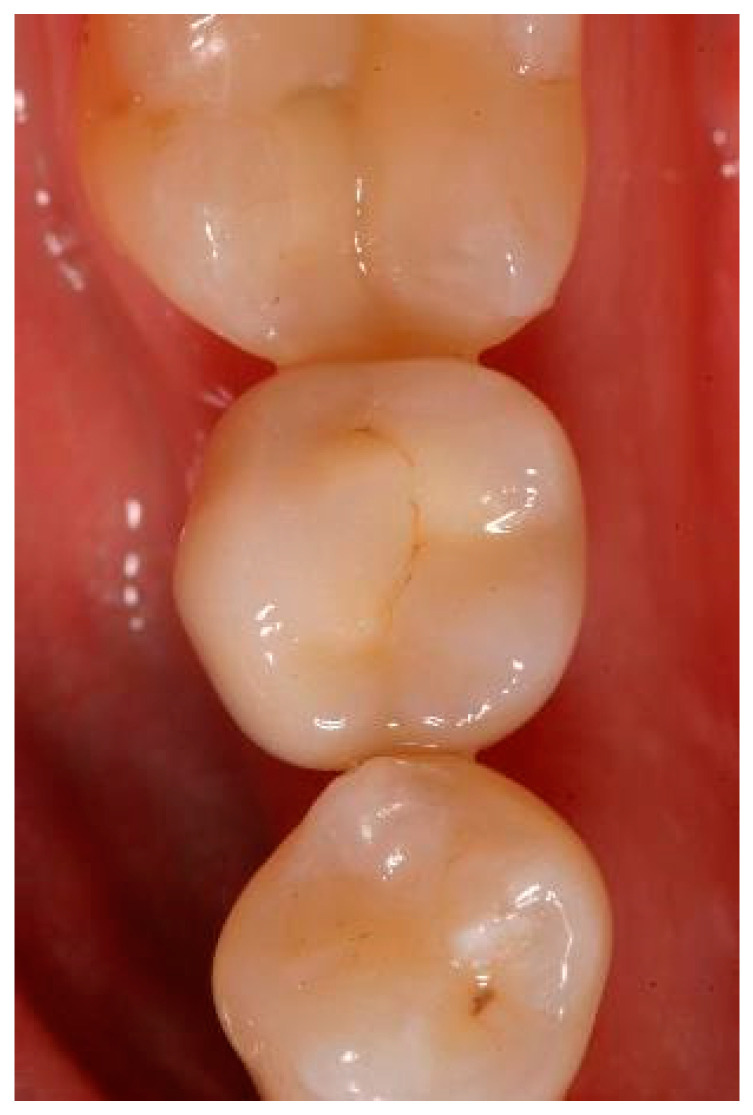
Situation after crwon cementation. Occlusal view.

**Figure A2:**
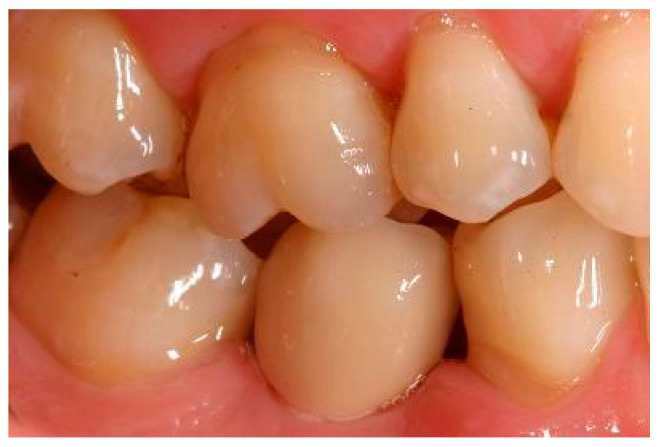
Situation after crown cementation. Lateral view.

**Figure A3:**
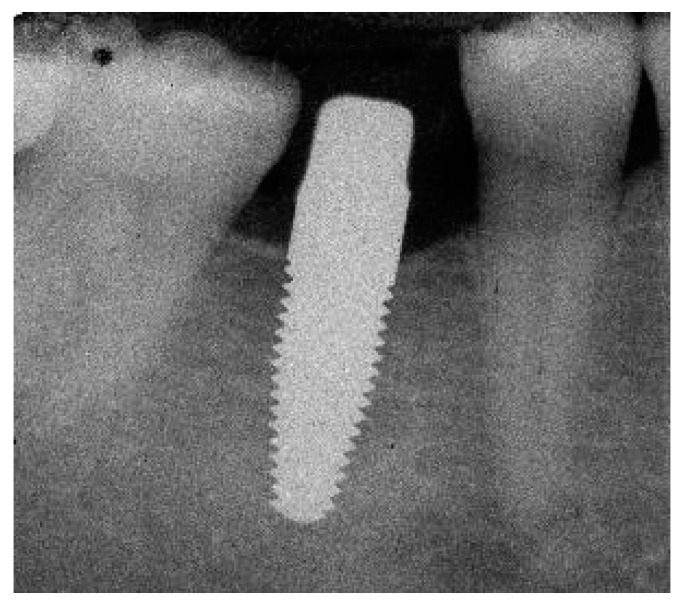
Radiograph of the perimplant bone situation before crown cementation.

**Figure A4:**
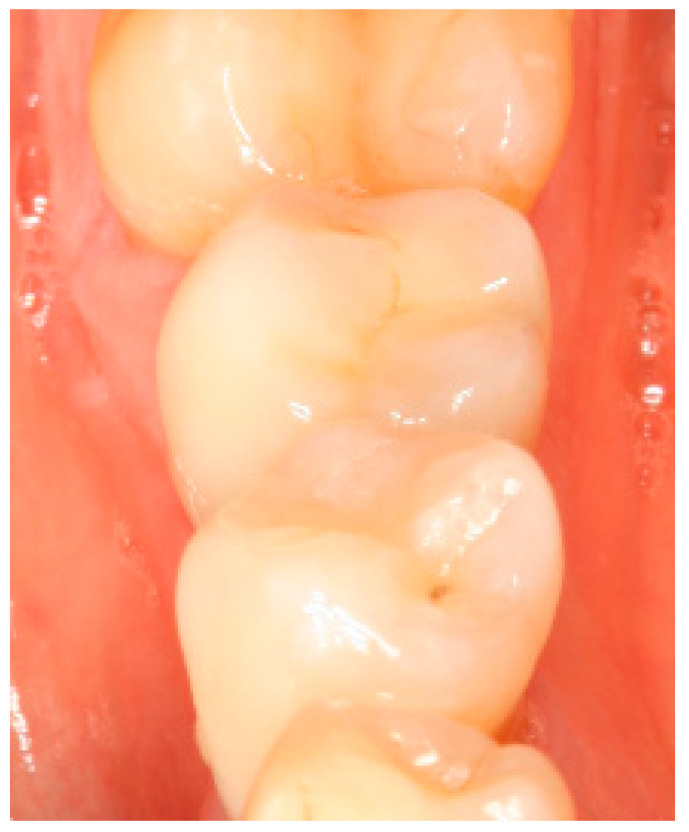
Clinical situation at the 1-year follow-up. Occlusal view.

**Figure A5:**
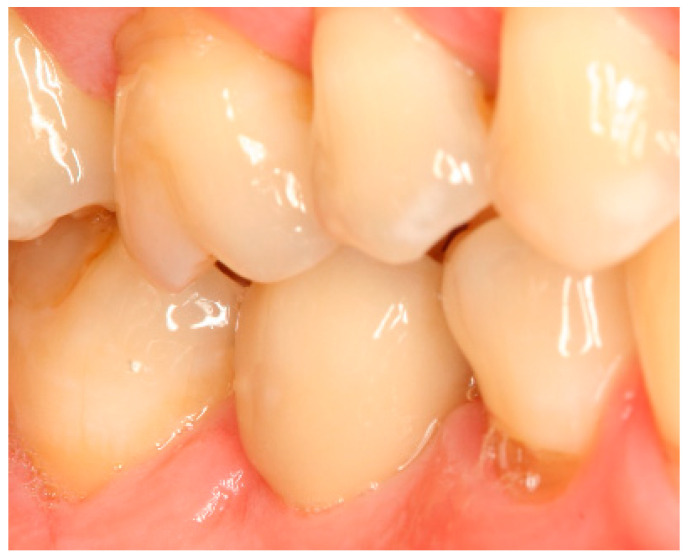
Clinical situation at the 1-year follow-up. Lateral view.

**Figure A6:**
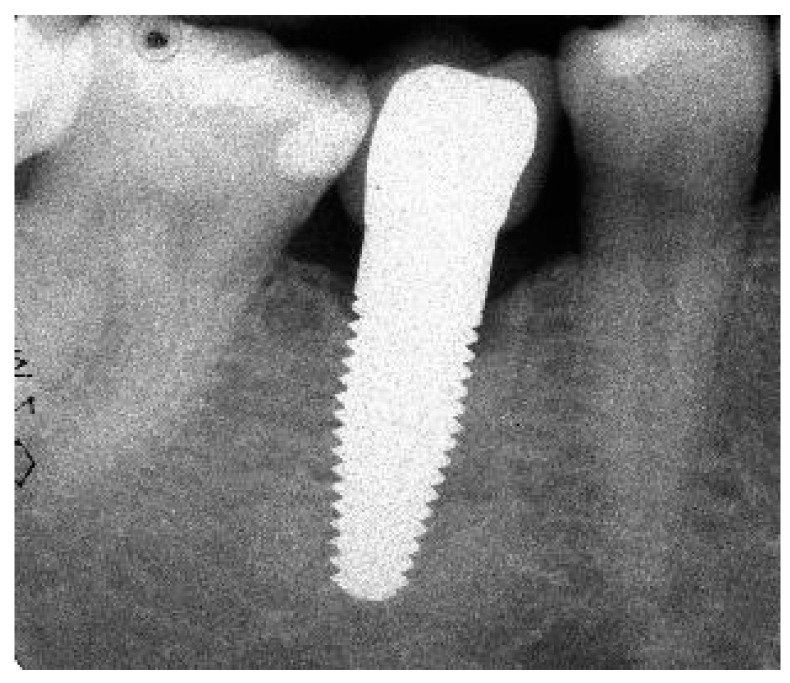
Radiograph of the perimplant bone situation at the 1-year follow-up.

**Figure A7:**
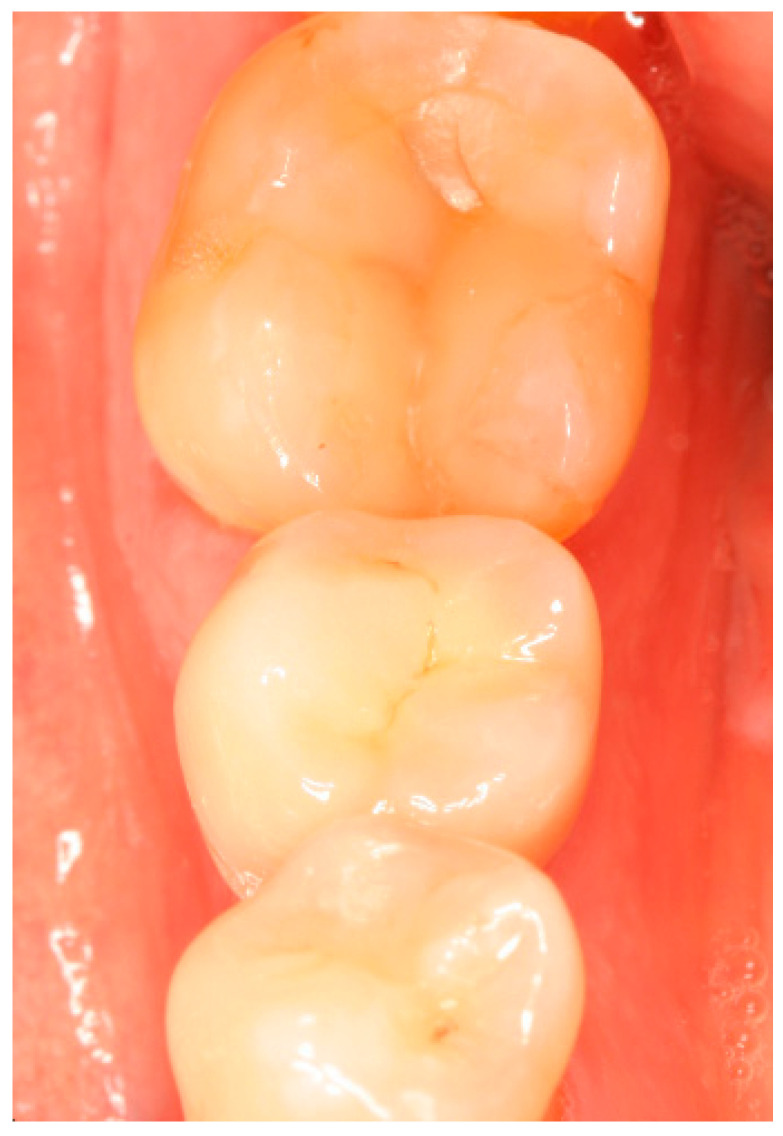
Clinical situation at the 3-year follow-up. Occlusal view.

**Figure A8:**
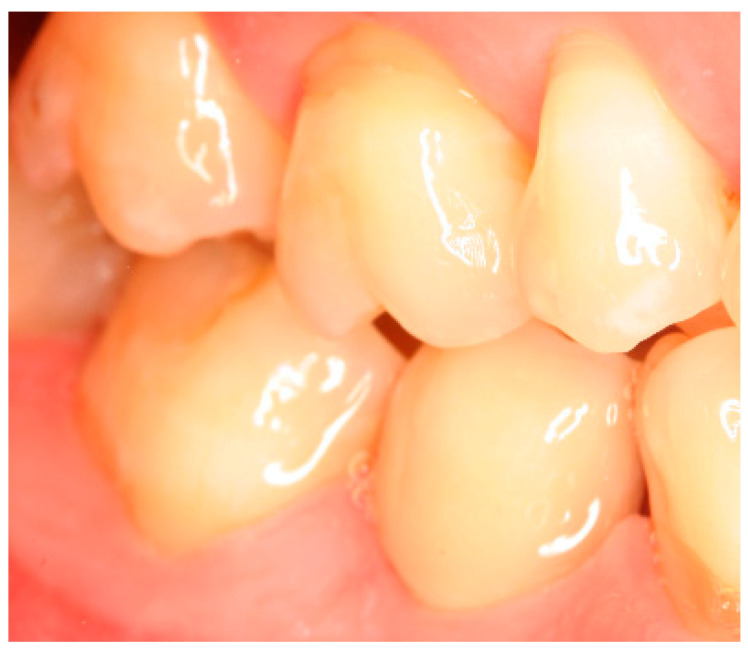
Clinical situation at the 3-year follow-up. Lateral view.

**Figure A9:**
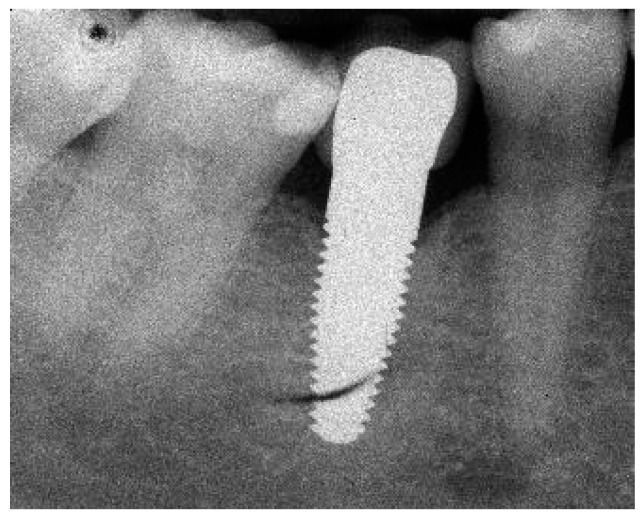
Radiograph of the perimplant bone situation at the 3-year follow-up.

**Figure A10:**
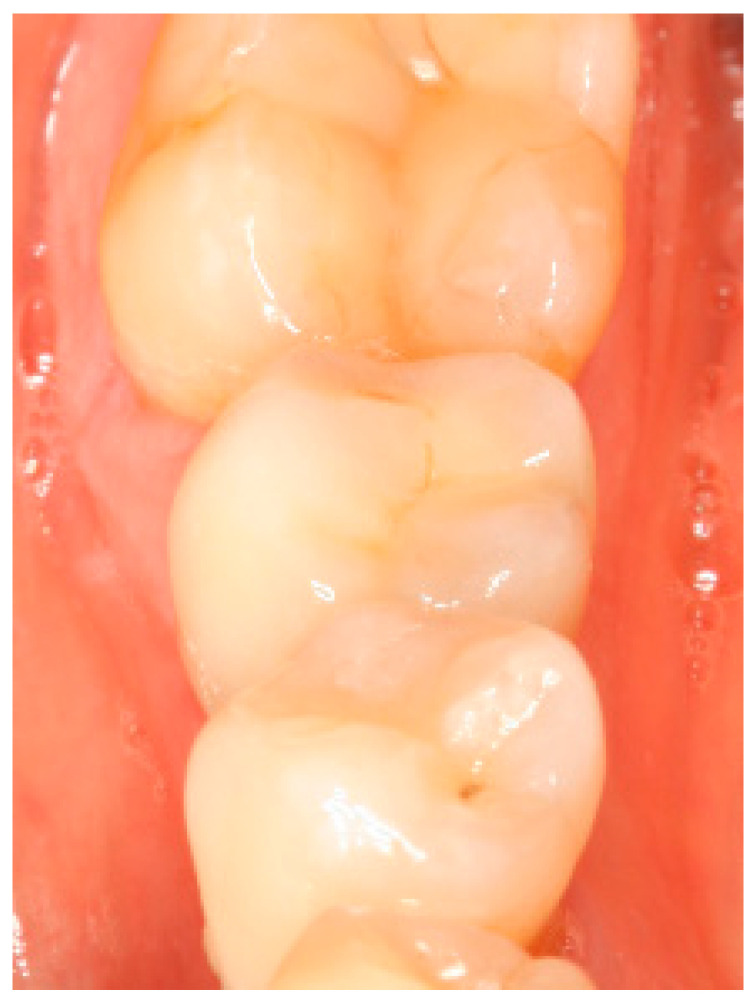
Clinical situation at the 5-year follow-up. Occlusal view.

**Figure A11:**
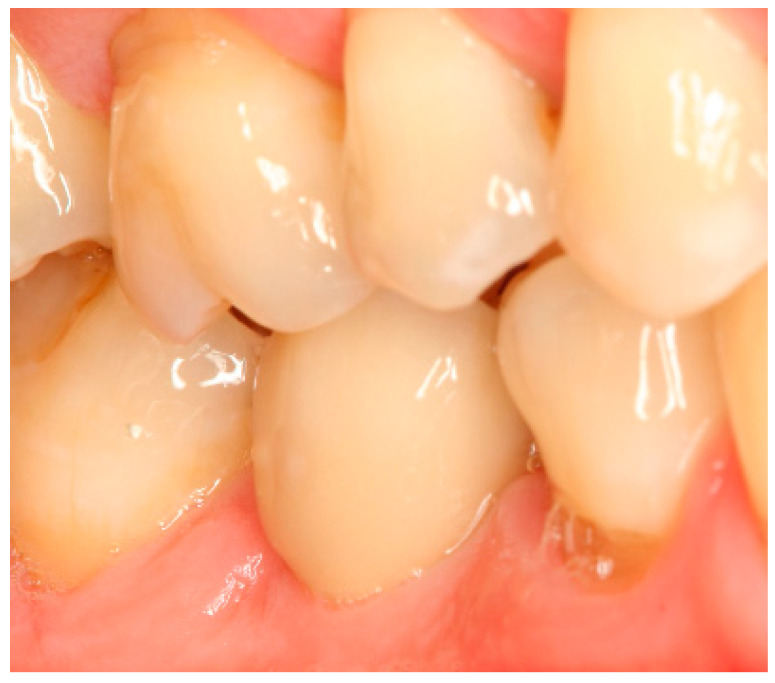
Clinical situation at the 5-year follow-up. Lateral view.

**Figure A12:**
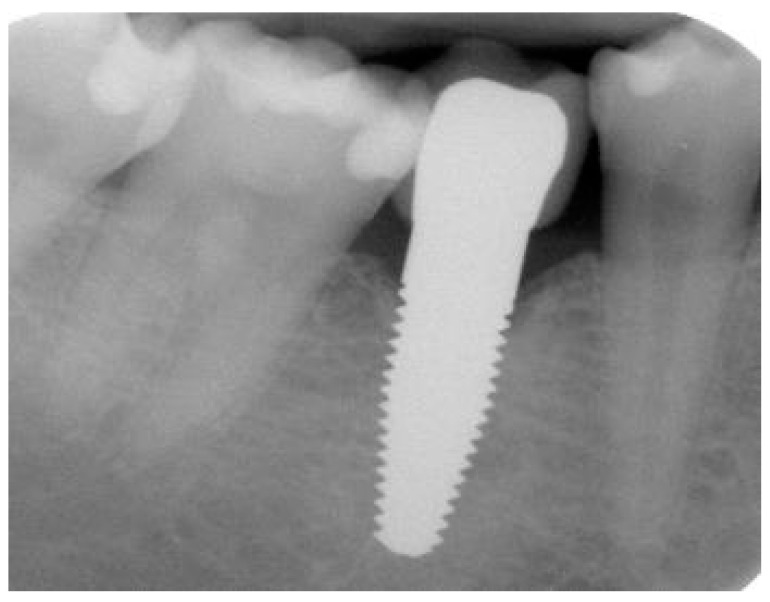
Radiograph of the perimplant bone situation at the 5-year follow-up.

**Figure A13:**
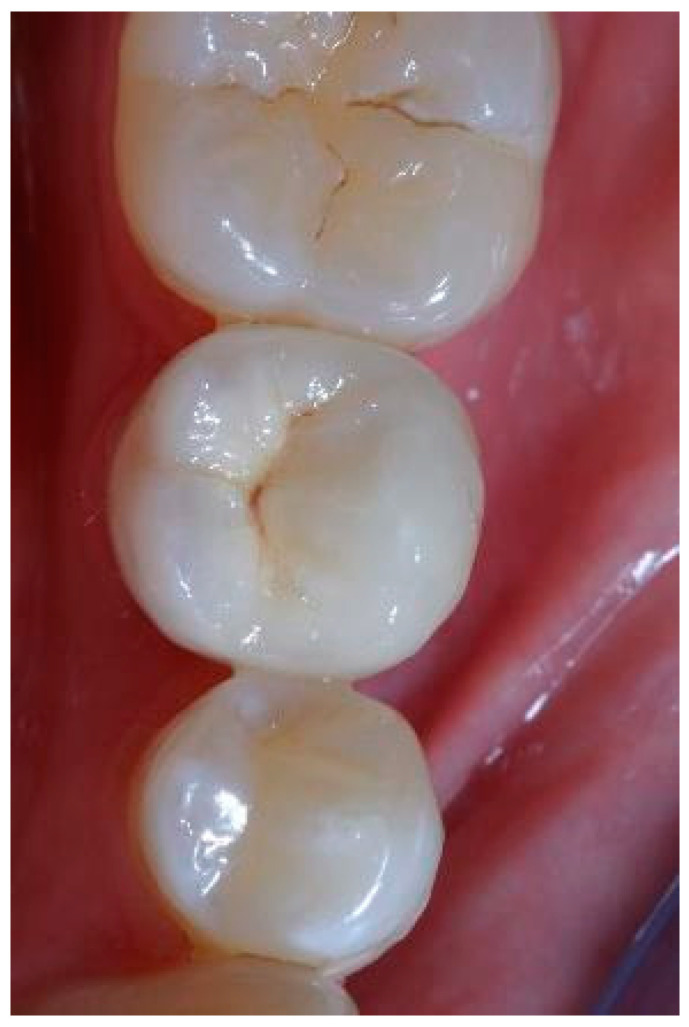
Situation after crown cementation. Occlusal view.

**Figure A14:**
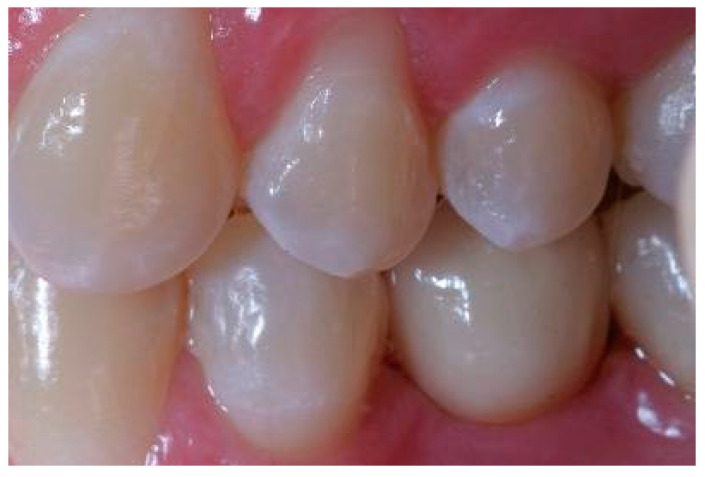
Situation after crown cementation. Lateral view.

**Figure A15:**
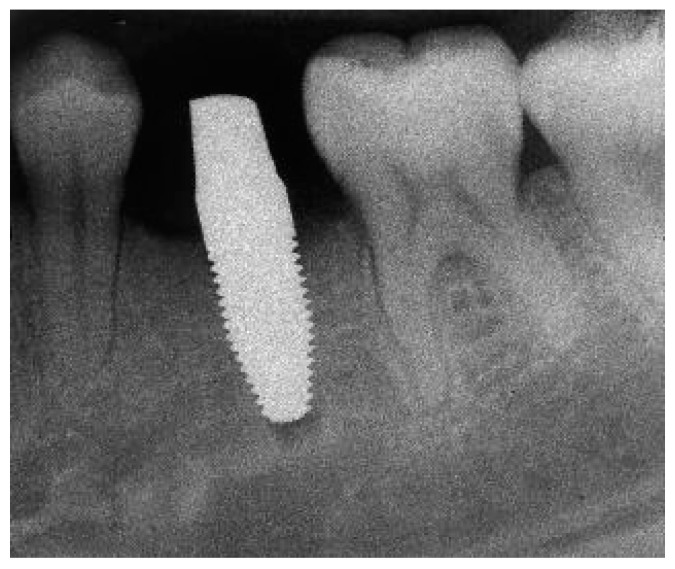
Radiograph of the perimplant bone situation before crown cementation.

**Figure A16:**
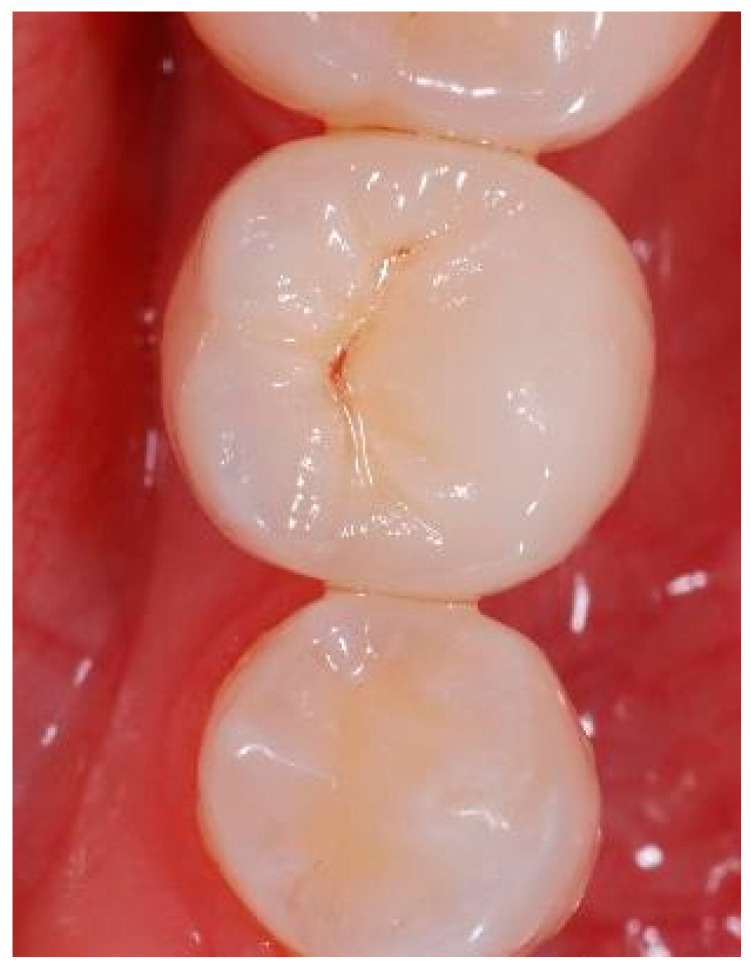
Clinical situation at the 1-year follow-up. Occlusal view.

**Figure A17:**
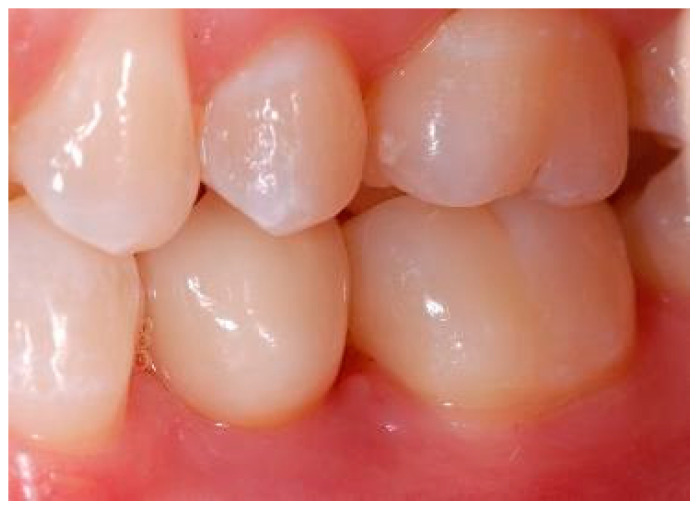
Clinical situation at the 1-year follow-up. Lateral view.

**Figure A18:**
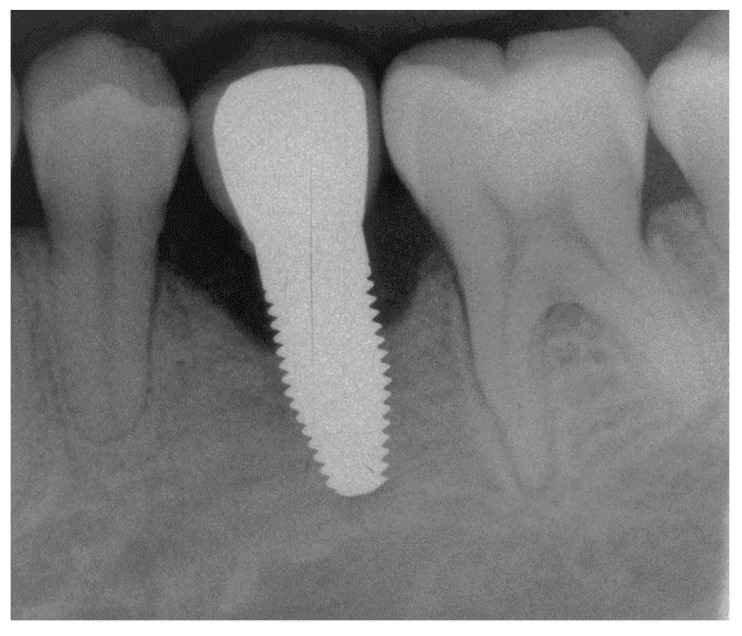
Radiograph of the perimplant bone situation at the 1-year follow-up. The bone loss is already advanced. Therapy: conventional subgingival cleaning with ultrasound and plastic tips; rinsing with chlorhexidine, reinforcement personal oral hygiene.

**Figure A19:**
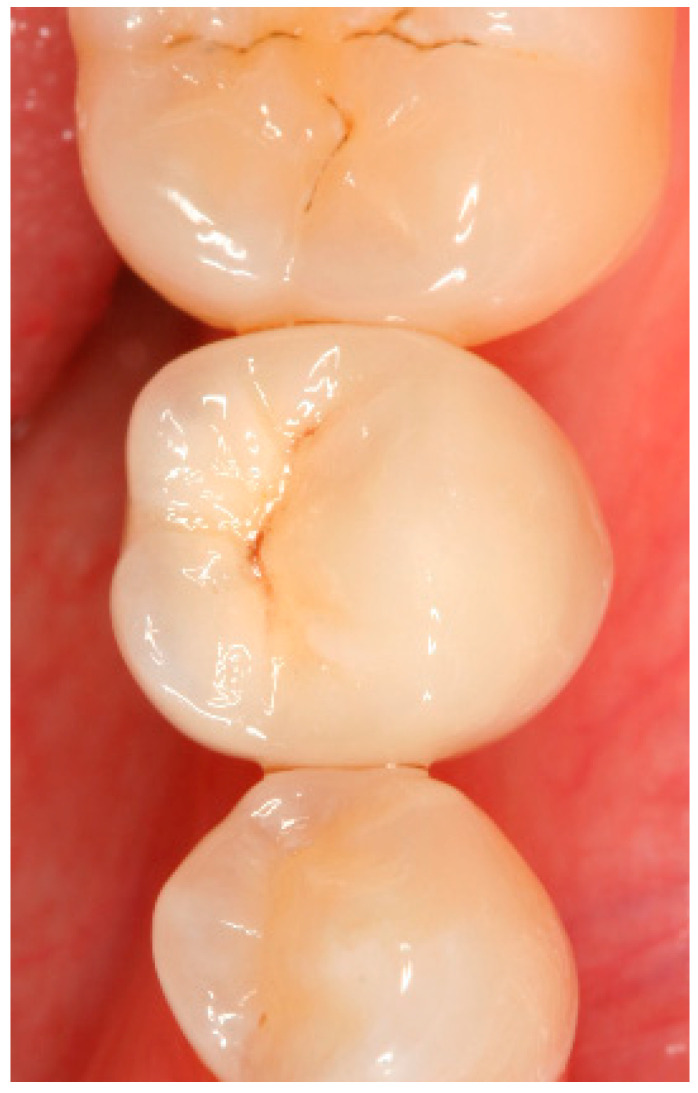
Clinical situation at the 3-year.

**Figure A20:**
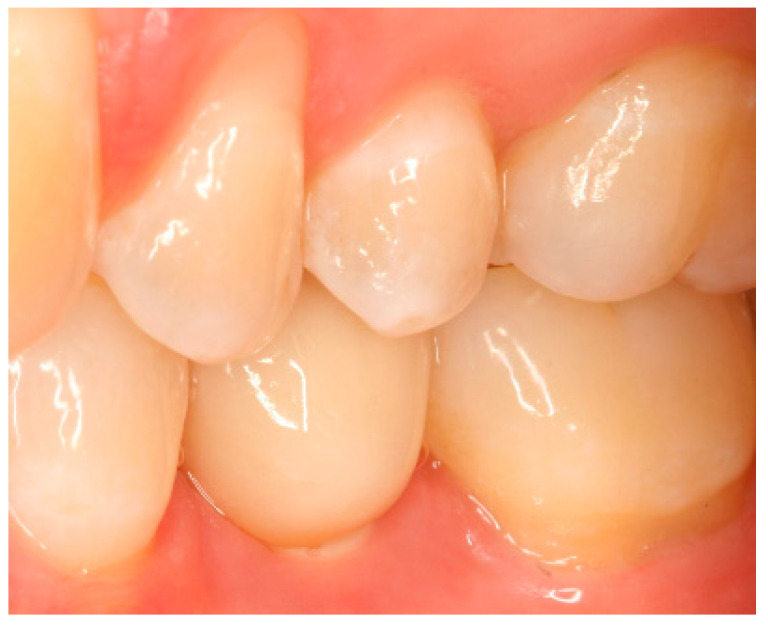
Clinical situation at the 3-year follow-up. Lateral view.

**Figure A21:**
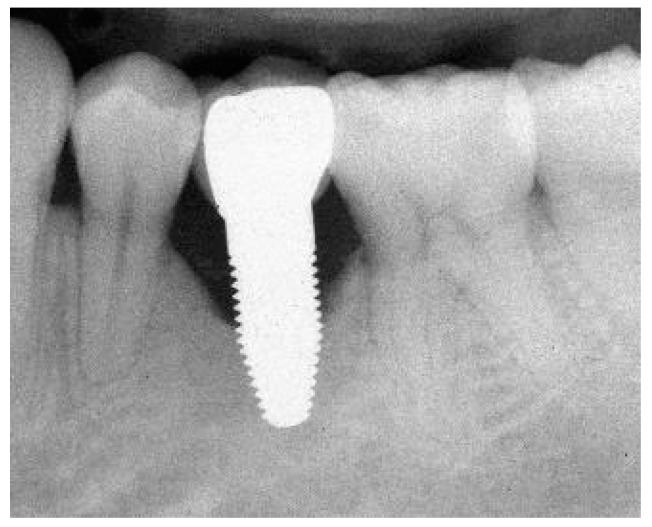
Radiograph of the perimplant bone situation at the 3-year follow-up. Compared to the 1-year follow-up, relatively stable bone situation.

**Figure A22:**
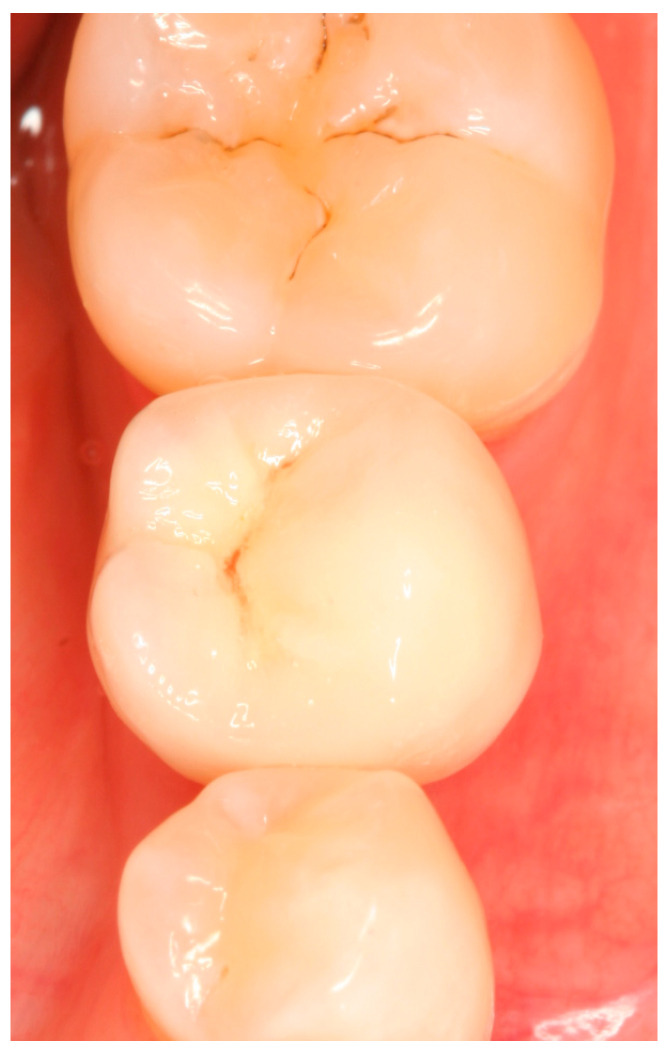
Clinical situation at the 5-year follow-up. Occlusal view.

**Figure A23:**
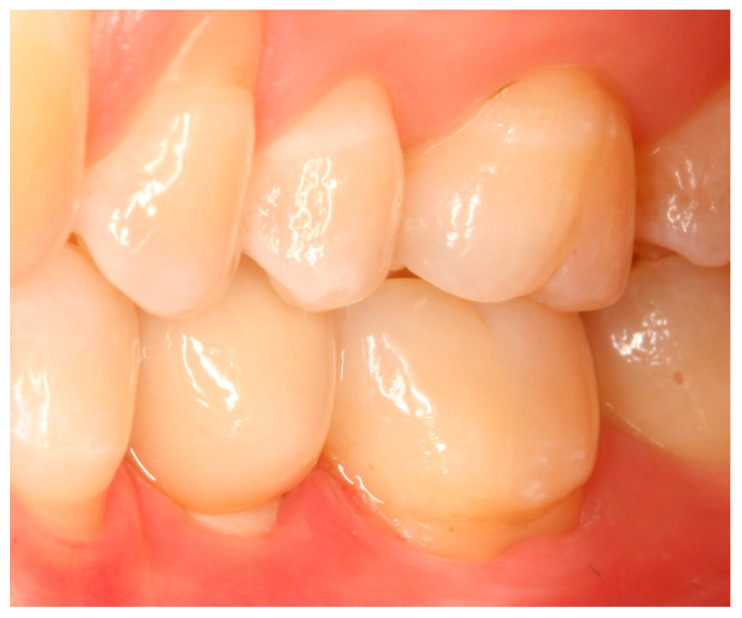
Clinical situation at the 5-year follow-up. Lateral view.

**Figure A24:**
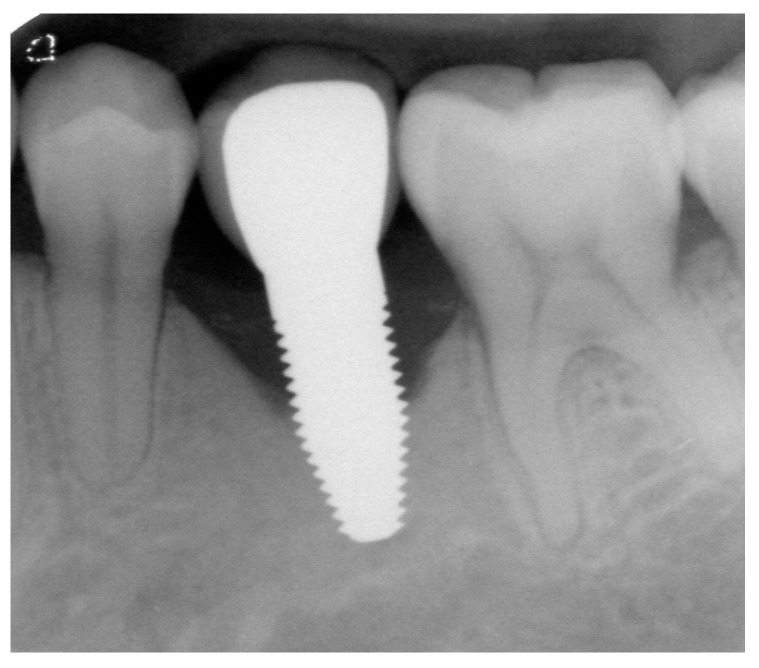
Radiograph of the perimplant bone situation at the 5-year follow-up. Aggravation of bone loss. Although, the clinical situation looks favorable, suppuration occurred. Patient requested removal of implant and no surgical intervention for cleaning.

## Data Availability

The datasets generated and analysed during the current study are available from the corresponding author on reasonable request.

## Appendix A. Exemplary Photographs and Radiographs from a Patient at Different Examination Time Points and a Positive Outcome

Patient A:

## Appendix B. Exemplary Photographs and Radiographs from a Patient at Different Examination Time Points and a More Negative Outcome

Patient B:
